# Experiences and views of people who frequently call emergency ambulance services: A qualitative study of UK service users

**DOI:** 10.1111/hex.13856

**Published:** 2023-09-17

**Authors:** Bridie A. Evans, Ashra Khanom, Adrian Edwards, Bethan Edwards, Angela Farr, Theresa Foster, Rachael Fothergill, Penny Gripper, Imogen Gunson, Alison Porter, Nigel Rees, Jason Scott, Helen Snooks, Alan Watkins

**Affiliations:** ^1^ Swansea University Medical School Swansea UK; ^2^ PRIME Centre Wales, Institute of Life Science Swansea University Medical School Swansea UK; ^3^ School of Medicine, PRIME Centre Wales, Division of Population Medicine Cardiff University Cardiff UK; ^4^ Public Contributor, c/o Swansea University Medical School Swansea UK; ^5^ Swansea Centre for Health Economics Swansea University Swansea UK; ^6^ East of England Ambulance Service NHS Trust Melbourn UK; ^7^ London Ambulance NHS Trust Greater London UK; ^8^ West Midlands Ambulance Service University NHS Foundation Trust West Midlands UK; ^9^ Welsh Ambulance Services NHS Trust Cwmbran UK; ^10^ Northumbria University Newcastle upon Tyne UK

**Keywords:** calling frequently, emergency care, emergency health services, prehospital, qualitative research, service user experience

## Abstract

**Introduction:**

People who call emergency ambulances frequently are often vulnerable because of health and social circumstances, have unresolved problems or cannot access appropriate care. They have higher mortality rates. Case management by interdisciplinary teams can help reduce demand for emergency services and is available in some UK regions. We report results of interviews with people who use emergency ambulance services frequently to understand their experiences of calling and receiving treatment.

**Methods:**

We used a two‐stage recruitment process. A UK ambulance service identified six people who were known to them as frequently calling emergency services. Through third‐sector organisations, we also recruited nine individuals with healthcare experiences reflecting the characteristics of people who call frequently. We gained informed consent to record and transcribe all telephone interviews. We used thematic analysis to explore the results.

**Results:**

People said they make frequent calls to emergency ambulance services as a last resort when they perceive their care needs are urgent and other routes to help have failed. Those with the most complex health needs generally felt their immediate requirements were not resolved and underlying mental and physical problems led them to call again. A third of respondents were also attended to by police and were arrested for behaviour associated with their health needs. Those callers receiving case management did not know they were selected for this. Some respondents were concerned that case management could label frequent callers as troublemakers.

**Conclusion:**

People who make frequent calls to emergency ambulance services feel their health and care needs are urgent and ongoing. They cannot see alternative ways to receive help and resolve problems. Communication between health professionals and service users appears inadequate. More research is needed to understand service users' motivations and requirements to inform design and delivery of accessible and effective services.

**Patient or Public Contribution:**

People with relevant experience were involved in developing, undertaking and disseminating this research. Two public contributors helped design and deliver the study, including developing and analysing service user interviews and drafting this paper. Eight public members of a Lived Experience Advisory Panel contributed at key stages of study design, interpretation and dissemination. Two more public contributors were members of an independent Study Steering Committee.

## 1 INTRODUCTION

Media attention and public concern about unsustainable demand for emergency ambulance services are high because call volumes steadily increase and long response times adversely impact patients, leading to calls for additional resources.^1^, ^2^, ^3^, ^4^, ^5^

Emergency ambulance services provide care for those with urgent and life‐threatening health conditions. However, fewer than 10% of calls relate to patients diagnosed with life‐threatening conditions.^1^ A small minority of patients make intensive use of emergency services,^1^, ^6^ often for unresolved health or social care needs.

UK ambulance services agree that people who make five or more calls per month should be classified as ‘frequent callers’, a threshold that may trigger particular service responses.^7^, ^8^ In London during 2014–2015, for example, 1622/1.7 million people meeting ‘frequent caller’ criteria received 49,534 ambulance attendances, costing £4.4 million.^1^, ^6^, ^9^ A similar story is repeated around the country where many services cannot match resources with demand.^6^, ^10^, ^11^

Research shows that people who make high use of emergency services also experience higher mortality rates.^12^ People who make frequent calls are often vulnerable,^6^, ^13^, ^14^, ^15^ more likely to: be from low socioeconomic groups; live alone; experience mental health challenges including self‐harming behaviour; live with chronic conditions; and have increased chances of falling.^16^, ^17^ Some studies suggest that they may have unresolved problems or call frequently because they are not aware of an alternative pathway to access appropriate care.^6^, ^16^, ^17^, ^18^, ^19^, ^20^, ^21^ A 2022 review identified only one qualitative paper—from Canada—reporting experiences of people making frequent emergency ambulance calls.^22^

Management of people who make frequent calls to ambulance services is a priority for United Kingdom (UK) ambulance services.^23^, ^24^, ^25^, ^26^ Approaches can include clinical triage in call centres, ‘hear and treat’ or multidiscipline working, because ‘people with mental health conditions have frequent crises and require a collaborative approach across the public service to ensure they are getting the right support’.^4^ UK commissioners require ambulance services to have management strategies in place for people who call frequently.^12^, ^14^, ^15^

Several ambulance services have initiatives to manage patients who call emergency services frequently, although approaches can vary across and within services^27^, ^28^ from ‘within‐service’ flagging and efforts to discourage further calls,^29^ to partnership through multidisciplinary cross‐sector team meetings in a case management approach.^6^ This aims to identify, share and manage patients collaboratively between commissioning, acute, primary, secondary and charitable health and social care providers and ambulance service staff.^28^ Effectiveness of clinical case management for people who use services frequently is uncertain, affected by wider determinants of health and how it is implemented.^30^, ^31^

We conducted a mixed methods study (STRategies to manage Emergency ambulance Telephone Callers with sustained High needs—an Evaluation using linked Data [STRETCHED])^32^ to evaluate effectiveness, safety and efficiency of case management approaches to the care of people who frequently call the emergency ambulance service and gain an understanding of barriers and facilitators to implementation (NIHR reference number 18/03/02).

In this paper, we report results of our qualitative strand exploring the views of patients who use emergency ambulance services frequently. Such patients may have experienced, or are likely to experience, case management if the initiative is available in their region. Patient experience is used in the NHS to identify strengths and weaknesses of healthcare delivery, drive quality improvement, inform commissioning and promote patient choice and patient‐centred care.^23^, ^25^, ^26^, ^33^, ^34^, ^35^, ^36^, ^37^ Our study aimed to understand health and care service users' experiences, generating evidence to inform commissioning, policy and practice development.

## 2 METHODS

### 2.1 Participants

We used a two‐stage recruitment process. Initially, we invited staff from four UK ambulance services to identify eight individuals 18 years and over from each ambulance service (*n* = 32) who received, or were eligible to receive, case management following their frequent use of emergency ambulance services. Our purposive sampling approach aimed to obtain a wide range of perspectives and experiences from individuals of diverse ages, backgrounds and ethnicity and with varied experiences of case management.

One ambulance service that operated case management was able to undertake this approach and recruited six participants. Two Frequent Caller Leads from two areas of that ambulance service purposively sampled people known to them. They contacted them twice, before and after providing an information sheet, to seek informed consent to an interview.

When staff in the other three ambulance services were unable to invite individuals because they or the case management team judged them too vulnerable to participate, we developed a second recruitment process. Public contributors were involved in discussing and agreeing the amended approach. We wanted their insight and experience to reduce any risk to the sample population, in light of the ambulance services' comments. In particular, they identified possible recruitment routes and helped draft and design recruitment information. They also circulated finalised information through their networks as part of the recruitment process. We gained ethical approval for our amended approach. Through third‐sector organisations, we sought individuals with health and care experiences reflecting the characteristics of people who call frequently,^6^ whether or not individuals believed they had received case management. We recruited these individuals through groups including the National Users Survivor Network, National Voices, the British Lung Foundation, the British Heart Foundation, National Centre for Mental Health cohort, which distributed through their newsletters and email groups to reach a diverse range of participants and perspectives. Over a 6‐month period, we also shared information through social media (regular Twitter postings and emails) and provided contact details for people who wished to take part. Those who expressed an interest received a study information sheet and consent form. All nine individuals who expressed an interest and received information were included in the study. Data collection ran from October 2019 to December 2021 (stage 1) and May to October 2022 (stage 2).

All interviews were by telephone or Zoom and took about an hour. Consent was reconfirmed verbally before interviews began. All participants were sent a £20 gift voucher to thank them for their participation.

### 2.2 Data collection

To gain a rich picture of patient experience and circumstances, we developed interviews with input from public contributors involved in our study. (see Supporting Information: File 1 for interview guide.) This approach provided an opportunity for patients to frame their own narrative about their circumstances, experiences and views regarding their needs, service use and care received, including any case management intervention. We also explored terminology.^38^, ^39^ A. K., A. P. and A. T. conducted interviews. With respondents' consent, we audio‐recorded and transcribed all interviews. All identifiable data were removed during transcription.

### 2.3 Qualitative analysis

We formed an analysis team of three qualitative researchers and two public contributors. Both public contributors had previous experience of analysing data through other public involvement work and their educational qualifications and did not require further training. We used a data‐driven thematic approach to analysis which generated themes from the implicit and explicit ideas within participants' accounts.^40^ We followed the six stages of analysis described by Braun and Clarke^41^; data familiarisation, generating initial coding, searching for themes, reviewing themes, defining, naming themes and producing a report. We assessed for data saturation^42^, ^43^, ^44^, ^45^ during analysis to see whether new themes emerged. We discussed emerging codes and themes within the analysis team and checked with the wider research team and public members of a Lived Experience Advisory Panel supporting the study. All transcripts were reviewed by at least two members of the analysis team before initial discussions and further review by the main author who then shared draft themes and write‐ups for comment. To ensure the iterative process was accessible for everyone, we did not use software such as NVivo but made and shared notes on word documents. Our discussions did not lead to disagreements, rather they enabled us to share different interpretations and insights to refine our understanding of service users' experiences. For example, public contributors highlighted how frequently service users felt ignored and unheard by service providers and the judgement implied in the way that professionals reacted to them. We have all agreed on the interpretation presented in the results and discussion sections of this paper. Our approach provided the analysis team with experience‐based expertise alongside research‐based expertise to strengthen insight and interpretation of the data.^38^, ^39^, ^44^, ^46^

### 2.4 Reporting

We report results according to themes identified in the data. We selected quotations to be representative of respondents' comments, unless otherwise stated. We identify respondents as groups 1 and 2 and with a unique number (e.g., Grp1‐1, Grp2‐1).

### 2.5 Public and patient involvement

Public contributors B. E. and P. G. were involved in developing, undertaking and disseminating all aspects of the STRETCHED research, including the qualitative strand reported here. They were research co‐applicants and also active members of the multidisciplinary Research Management Group. This group, made up of co‐applicants and study advisors, included paramedics, ambulance service managers, public contributors, methodologists, statisticians, health economists and research staff, and was responsible for study implementation. B. E. and P. G. also analysed interviews, developing themes, guiding interpretation and reviewing draft results with A. K., B. A. E. and A. P. These were then reported back to the Research Management Group for comment and synthesis in the full study findings. In preparing this paper and other reports, they paid particular attention to the language used. They were sensitive to implied bias, judgement and privileges that influence how people might be described and perceived by themselves or others. We also established a Lived Experience Advisory Panel of eight public contributors, chaired by P. G., which met at key stages of the study to provide in‐depth input to study design, interpretation and dissemination. All public contributors had relevant lived experience to inform their involvement in the STRETCHED study [32, 38, 38 = 9, 46]. Their experience‐based expertise included: using emergency ambulance services or supporting family members to do this; knowledge of the health conditions experienced by people who call 999 services frequently; experience of the professional services provided to support people who call 999 services frequently (Table 1). We supported our public contributors to collaborate as equal members of the research team throughout. In addition, we recruited two public contributors to the independent Study Steering Committee.^46^, ^47^

**Table 1:** Role of public contributors.

Public contributor	Ambulance service	Role
*Research co‐applicant and RMG member*		
01	A	Design and submission of research proposal; management, delivery and dissemination of research; analysis and presentation of qualitative data
02	A	
*Study Steering Committee member*		
03	A	Oversight and advice to the RMG and research co‐applicants
04	A	
*Lived Experience Advisory Group member*		
01 (LEAP Chair; reporting between LEAP and RMG)	A	In‐depth input to: study design including commenting on interview questions and participant recruitment information study interpretation including commenting on early analyses and draft results study dissemination including feedback on presentations and reports
02 (reporting between LEAP and RMG)	A	
05	B	
06	B	
07	B	
08	C	
09	D	
10	D	

Abbreviation: RMG, Research Management Group.

## 3 RESULTS

We interviewed 15 service users. Six respondents were identified by an ambulance service as eligible to receive case management. The other nine responded to information about the study, distributed by third‐sector organisations and on social media. We present our results in three parts. In the first two sections, we report views and experiences of each respondent group about making frequent calls to the emergency ambulance service and case management. Third, we review common themes across their experiences.

*Group 1*: People eligible for case management from their local ambulance service.

### 3.1 Characteristics of respondents

This group was recruited in line with our initial qualitative plan. Characteristics and calling patterns are shown in Table 2.

**Table 2:** Characteristics and calling patterns of people eligible to receive case management from their local ambulance service (group 1).

Respondent	Health	Calling pattern	Calling trigger	Response received	Perceived need	Case management status
1‐01	Heart disease, IBS, asthma, depression, anxiety, panic attacks	Called 999 frequently but less now on different medication	Breathing difficulties caused by asthma, heart issues or panic attacks	Ambulance attends, calms respondent, stays until feeling recovered	Different housing near father, away from home shared with deceased mother	Eligible
1‐02	Epilepsy and paranoia	Unclear how often called for ambulance	Unclear—combination of condition symptoms causing breathing difficulties	Ambulance attends. Checked and left or conveyed to hospital against respondent's wishes	Has a range of unmanaged health problems but no formal support to address them	Eligible
1‐03	Mental health diagnosis; self‐harms	Appears to call frequently but no details of how often. Calls ambulance, police and OOH	Becoming upset and panics. Triggers can be disagreements or the unexpected happening. Almost always calls when spouse is not at home	Attended by ambulance or police. Conveyed to hospital if needing further treatment or surgery	Has weekly contact with GP and monthly psychiatrist plus uses a mental health freephone number	Eligible
1‐04	Aneurysm, asthma, leg injury, depression and HIV	Unclear but seems known to ambulance and police	Breathing difficulties linked to a chronic lung condition; poor mental health; alcohol use; anxiety about family members' health	Attended by police and ambulance. Neighbours also make emergency calls. Unclear how often taken to hospital	A support worker. Would like to return to studies and get paid or voluntary work	Eligible
1‐05	Emotionally Unstable Uersonality Disorder diagnosis; has suicidal thoughts and self‐harms	Calls mental health helpline most days. Helpline calls 999 if consider respondent is likely to harm self or others	Calls are made by other people because of psychotic episodes	Police and ambulance often attend. If taken to hospital, is discharged after a few hours.	Feels not listened to, misdiagnosed and mismedicated. Wants a support worker physically present when in crisis to properly assess the situation and avoid needing to call 999	Eligible
1‐06	Congenital heart condition, ADHD, anxiety; has self‐harmed	Can call up to 30 times a month, always at night	Chest pains and heart palpitations, exacerbated by anxiety. Has congenital heart condition, mental health issues, anxiety and low trust of others	Ambulance attends because of symptoms. Police can be called if respondent is violent. Conveyed to hospital if consents but unwilling to travel in the ambulance without partner or sister. Usually checked and discharged from hospital.	Feels services have no understanding of respondent's heart health experience so cannot manage it. Wants to be kept in for prolonged observation. Wants medical support worker; distrusts social and probation services	Eligible

Abbreviations: ADHD, attention deficit hyperactivity disorder; GP, general practitioner; HIV, human immunodeficiency virus; IBS, irritable bowel syndrome; OOH, out of hours.

These respondents all experienced mental health needs, usually alongside or exacerbated by physical symptoms linked to other conditions including heart disease, epilepsy and HIV. One confirmed a diagnosis of autism and attention deficit hyperactivity disorder (ADHD), while another said they had an eating disorder. These respondents were without paid work nor had completed college courses and five lived alone. Two had been homeless as teenagers, two recalled their parents were drug addicts and another's was addicted to alcohol, while one respondent was raised in a foster family and care home. Three said they used illegal drugs or alcohol to relieve their symptoms. Although they experienced acute health needs which prompted 999 calls, their circumstances reflected a complex interaction of factors including long‐term health needs, childhood trauma, bereavement, homelessness and unstable mental health.

### 3.2 Experience of calling and receiving emergency ambulance care

All respondents provided accounts of their interactions with emergency health services, which were often vivid for the level of dissatisfaction and despondency their reports conveyed. They said they called 999 for emergency help as a last resort, when access to other support was not available or because their situation seemed to overwhelm them. One called a mental health helpline and another called 111 instead of 999 and it was these call takers who alerted the emergency ambulance and police when they felt the situation warranted further action.

I don't think I can manage it … like last night I thought my heart was going to stop. I went cold to the point where I thought there was—[inaudible 0:07:16], so my chest just felt weird to what it would normally do. (Grp1‐06)

Respondents did not appear to know whether they were receiving case management. Respondents said they relied on family, friends or neighbours because they felt alone and unsupported in their mental and physical health needs. Three said they self‐medicated with alcohol or drugs. Their comments conveyed a strong sense that they felt unheard and ignored. One respondent, many months into an application to be rehoused, said she had a letter of support from the ambulance service and was helped by the charity MIND. She was the only person to mention the ambulance service outside a 999 response. One person said she was in regular touch with a mental health charity, saw her general practitioner (GP) every week and received a monthly call from a community psychiatric nurse. Despite these contacts, she was unable to cease making 999 calls when experiencing panic attacks, and self‐harming. Her husband could not reassure her either, she said.

he tries to put me off it, calling, he tries to talk me out of it because there's more serious cases than you, so don't you dare, but I—if I—it gives in—I give in then and say, ‘Look, I've got to’ (Grp1‐03)

Another said she had a support worker and management plan but these were imposed on her and did not meet her needs when experiencing crisis.

I do have twenty‐four hour support workers where I live but again the management plan tells them they're not allowed to come and support me when I have suicidal feelings, or I've self‐harmed, or I'm going to self‐harm. They're not allowed to support me, so I don't understand who I'm meant to turn to. (Grp1‐05)

Respondents said they felt ‘criminalised’ by the way they were treated by emergency services. Four of the six were attended by police officers when calling for an emergency ambulance. One said she was arrested for violent behaviour linked to a psychotic and self‐harming episode. Another was warned for wasting police time with regular 999 calls. A third recalled escaping from police and security guards when he discharged himself from hospital. These accounts, made within longer reports of their emergency calling, suggested that police involvement was a normalised element of their experiences. One respondent also said he was threatened by the ambulance staff for refusing treatment during a mental health crisis because of his fear of hospitals.

It's basically like refusing to be arrested, isn't it? It's like they threaten me for refusing to go there, isn't it? They start mentioning things like, ‘Oh, you won't get benefit anymore because you're not taking our advice and our services’. And suddenly your back's, like, isn't it, it's just I'm petrified of hospitals. (Grp1‐02)

It was clear during these interviews that some respondents were afraid of hospitals and health services, distrustful of therapies such as counselling, which required them to regularly engage and communicate and also nervous of social and probation services. Even though they were in emotional and physical pain, fear appeared to shape their interaction with health providers and prevent them from taking care of themselves.

They also felt angry and disillusioned that their problems were not acknowledged and addressed.

I'm just pissed off with like going back and forward to the hospital to these meetings, when they can just keep me in there, just like they do with anyone else, and see what the situation is … they would see my issue comes on every now and then, and my body feels like it's not working. They think I'm just being lazy. (Grp1‐06)

If they were offered treatment, it often did not fit their needs nor were they able to keep appointments or make follow‐up arrangements. One respondent specifically asked for support so he did not need to rely on family but said he was still waiting to receive help.

I'm looking to get another support worker. I just don't know how to go about it…

I was meant to be going for counselling before, a long time ago, but I never heard nothing about it. (Grp1‐04)

They usually wanted input at times of crisis rather than receive a programme of contacts at a set time, which may not coincide with their symptoms. These respondents often felt their needs would be resolved by nonhealth interventions such as better housing, training, paid or voluntary work.

all I want to do at the moment is get my own place, so my mental health issues can drop. (Grp1‐01)

*Group 2*: People who identified as making frequent or regular 999 calls.

### 3.3 Characteristics

There were nine respondents in this group, recruited through social media and the third sector. Three respondents reported mainly mental health problems, which caused suicidal and self‐harming behaviour. One was a carer for his sister with physical and mental health diagnoses. One respondent and a family group—mother, father and daughter—reported physical illnesses. The family came to the United Kingdom and the daughter translated for her parents during NHS contacts and the interview. One respondent said a family member mostly made the emergency call because he was not usually conscious, while another said 111 call handlers called the emergency ambulance. One respondent said he was treated by the High‐Intensity User Group within his ambulance service but only found this out some years later, after his emergency calling ceased.

A summary of respondents' characteristics and calling patterns is shown in Table 3.

**Table 3:** Characteristics and calling patterns of people who identified as making frequent or regular 999 calls (group 2).

Respondent	Health	Calling pattern	Calling trigger	Response received	Perceived need	Reported case management status
2‐01	Mental health diagnosis; has suicidal thoughts	Respondent called 111 mainly. Called frequently over a 15‐month period, sometimes up to 44 times a month	Called when needing support and feeling suicidal	No treatment. Call handlers hung up after being flagged as frequently calling	Regular mental health support. Crisis arose when this support was removed	Received, in the past
2‐02	Mental health diagnosis; has attempted suicide	Respondent called 999 frequently over an 18‐month period	Called when experiencing suicidal thoughts	Conveyed to ED but received no treatment until made an actual suicide attempt	Regular mental health support	Received, in the past
2‐03	ADHD	Family called 999 when found respondent unresponsive. Calling 999 was a long‐term situation but the frequency of calls was unclear	Used alcohol or drugs to help manage mental health crises including severe ADHD	Perceived as a drug addict since used amphetamines to manage ADHD	Prescription version of ADHD and mental health support	Unclear
2‐04	Carer to elderly sister who is frail, has rheumatoid arthritis and bipolar disorder	Respondent calls 999 once or twice a month for help when sister falls	Needs help to lift sister when she falls or is confused by medication	Paramedics lift sister and make her comfortable. Sister conveyed to hospital if experiencing a mental health relapse	Weekly carer support so respondents can have a regular break from caring routine	Not receiving/not available
2‐05	Severe chronic asthma	Respondent calls 999 once or twice a month if experiencing severe asthma attack out of GP hours	Asthma attack which does not respond to the multiple treatment options respondent has available and: GP not available; no one available to take him to ED	Paramedics administer required medication. In rare and most extreme cases of breathing difficulties, has been admitted to hospital overnight	Out‐of‐hours access to medical support to administer rescue medication	Not receiving/not available
2‐06, 2‐07, 2‐08	Parents have problems with heart, kidney, ears, knees and dental issues	Respondent called 999 or 111 once or twice a month when the family newly arrived in the United Kingdom but the frequency has declined as they are more familiar with the healthcare system and English language skills are better	Unable to see GP because no available appointments; OOH so no transport to pharmacist; perceives no alternative for range of health concerns and pain experienced by family members	Attended by ambulance and treated. One reported conveyance to hospital after three calls for symptoms	Ability to make appointment to see a GP (not necessarily same day)	Not receiving/not available

Abbreviations: ADHD, attention deficit hyperactivity disorder; ED, emergency department; GP, general practitioner.

### 3.4 Experience of calling and receiving emergency ambulance care

Respondents said their emergency call was made as a last resort. They, or someone calling on their behalf, genuinely believed the situation was urgent and they were a danger to themselves. Some respondents described steps they took to delay or avoid a 999 call. These included taking extra medication, waiting to see if the problem would resolve, contacting a GP or pharmacist or phoning a helpline.

It's only when it's absolutely necessary that I ring 999 and ask for ambulance … We do not make demands on any public services including ambulance service unless it's absolutely necessary. (Grp2‐04)

Respondent 01 realised he was flagged as a regular caller when call handlers deflected or terminated his emergency calls, made when feeling suicidal. He said his need for help was misinterpreted by emergency and mental health professionals and he felt labelled as a nuisance. When his case was taken on by the High‐Intensity User Group without his knowledge, no one listened to his perspective or provided the support he wanted. He said he felt ‘bullied’ and ‘dehumanised’ by health professionals.

it's brutal—it's not having the flag, it's how you're treated because the flag is there.

If they have a system whereby they want to say, anyone that's calling more than five times is a frequent caller, then I'm okay with that, put me up on the system as a frequent caller. But it's what you do with that flag—rather than have it as a label with a stigma attached that he's a nuisance, say we've got a vulnerable adult that's in distress and needing help. (Grp2‐01)

Respondent 02 also said the way her needs were dealt with was ‘brutal’ and undermined any sense of self‐worth. She said she felt rejected when repeatedly discharged from the Emergency Department because her suicidal thoughts had not resulted in an injury that needed treatment. She said people who made frequent emergency calls had unresolved problems.

if someone is a frequent caller they often have unmet needs of some kind. So, you know, it should be a flag to indicate someone has unmet needs and that should be acted upon. (Grp2‐02)

Respondent 03 echoed this impression of isolation and desperation, He said he feared that a system which identified frequent callers to 999 call handers could ‘blacklist’ people and stop them receiving necessary treatment.

Respondent 05 experienced severe asthma, which left him choking for breath if a flare‐up did not respond to his usual medication. Out of hours (OOH), he was unable to access the emergency treatment, which could resolve these attacks. An hour's drive to the Emergency Department was not feasible in this state and with very young children to care for, so he would call for an ambulance. But he was shocked by the off‐hand manner of 999 call handlers and waiting as long as 12 h with symptoms, which left him unable to breathe. As a regular caller, he queried how his needs were prioritised in a system, which made him feel like a piece of meat, not a human life.

I can't breathe through my nose, I can't breathe through my mouth, I get palpitations and I'm gasping for air. That's no less than a heart attack in my view. So, what priority am I? Am I a one or am I a ten? If I'm a two, after a heart attack, then how long should I wait, and how is that allocated? It's happened before where I've fainted and blacked out when I couldn't breathe … There's no empathy, there's no compassion. And I think some of the staff are just admin staff. They read off a script. It's passed like a sausage factory to somebody else. (Grp2‐05)

The family group and the carer called with specific illnesses and less urgent needs. The carer usually needed help to lift his sister or check her when her medication made her nonresponsive. He welcomed the idea of being identified by the ambulance service for additional support, seeing it as a way to remedy the uncoordinated and inadequate care he received from the health and social care sectors. He was of Asian heritage and was very committed to caring for a close family member. However, his greatest wish, as a carer, was for some brief periods of respite in a clearly precarious domestic situation.

With the ambulance crew being the first contact, I think it's an excellent method for them to be using their eyes and ears to observe what needs to take place and then they make the referral to this support team who will then come and assess the needs of the person … make a referral to more appropriate services. (Grp2‐04)

The family group said they were encouraged to use 999 services when newly arrived in the United Kingdom and not fluent in English and continued to seek emergency help, particularly if unable to get a GP appointment or access a pharmacist. They were full of praise for how their calls were addressed.

I think [999] is the best because if you have language, or you don't have language, they will still find a way to get contact with you, or to get the best care for you. (Grp2‐06)

### 3.5 Common themes

Across these two groups of respondents, there were common issues in their experiences. All said they called for emergency care as a last resort because they had no other routes to access help. Their perception of their situation was that it was precarious and their health posed a danger to their life, or to the life of the person they cared for. Respondent 1‐02 described his fear of dying and need for reassurance when experiencing a crisis.

I just want them to make sure I'm not on my deathbed. It's so easy for somebody to just lose their life, through something like epilepsy and stuff like that. So I get over‐paranoid, it plays in with the psychosis, and I can start fitting and it's terrible. (Grp1‐02)

Respondent 2‐05 vividly conveyed the sense of panic he experienced during an asthma attack and having nowhere else to turn.

I only call at the absolute latest, when I think I can't take it no more. That's the time I need the most support. There are times when I just can't breathe. I'm gasping for air. I've taken my pump and nothing's happened. I've taken the Ventolin, nothing's happened. Taken the Salbutamol, nothing's happened. The Montelukast, Azithromycin, I've taken all of that and I'm still gasping for air. … times no one's there, I can't get to a GP, I think I'm going to collapse, that's when I call 999, when it's absolutely critical, otherwise I don't want to waste my time or anyone else's. (Grp2‐05)

Except for the carer and family group, all respondents were deeply dissatisfied with the way they were treated and that the emergency issue remained unresolved. They said they felt ignored, judged, brutalised, rejected, dehumanised and threatened by the emergency services and clinicians who dealt with them. Instead of feeling flagged for tailored and coordinated support by health professionals, they felt labelled as troublemakers and nuisance callers. Even though some individuals received support—from clinical specialists, mental health teams, a third‐sector organisation or a GP— respondents felt isolated and powerless.

I've tried the GPs, and out of hours GPs, and solicitors, and other people that I've worked with that don't believe that I have it either but it's very hard to, like, tell the psychiatrist and stuff like that because, you know, they don't listen. That's part of the problem—That I don't get listened to. (Grp1‐05)

Respondents generally had long‐standing and complex care needs and felt demoralised and fearful after repeated contacts with health professionals had not reduced or cured their symptoms. They seemed to use the emergency services as a gateway to trying to access better care and resolve their problems but found this route barred by gatekeepers who seemed unwilling to address their needs.

if they were there to help you, they should listen to what the people say, give me a chance … I think they've got to hear how I feel. They can't feel what's going on in my body, I can, they need to have a little bit of leeway and let me do what I've got to do to prove it. (Grp1‐06)

*a period of regular help seeking which had failed at every point. And really where I hadn't known where to turn. And so the ambulance service had kind of been the last resort … Well I exhausted—I tried all other options and repeatedly got nowhere… (Grp2‐02)*

They felt they had run out of nonemergency options for help. Communication between them and care providers appeared to have broken down, ways that services were allocated or delivered did not work for them and they felt unsupported and unheard.

So I wasn't ever a person to call up begging for help in distress … my support was completely cut off and that was the trigger for me trying to call in services for other support … I was calling in both for support and to try and get a more long term solution. (Grp2‐01)

## 4 DISCUSSION

We found that people make frequent calls to emergency ambulance services as a last resort, when they perceive their care needs are urgent and other routes to help have failed. Those with the most complex health needs generally felt their immediate requirements were not resolved and underlying mental and physical problems led them to call again. A third of respondents were also attended by police and had been arrested for behaviour that was associated with their health needs. Those callers receiving case management did not seem aware they were identified for additional support by health services. Other respondents, asked to reflect on the case management approach, criticised it for labelling frequent callers as troublemakers and felt it could operate, in the words of one respondent, as a ‘blacklist’. One respondent, a carer, welcomed the possibility of integrating health and care organisations to improve support to vulnerable people.

### 4.1 Strengths and weaknesses

We recruited through an ambulance service and by distributing information on social media and through the third sector. This enabled us to include a range of views and experiences, including: people living in England and Wales; people who currently or previously received case management and also some who had not received the intervention: individuals experiencing varied and multiple health conditions, demographic, economic and social circumstances. Additionally, one respondent was a carer and from an Asian background and three were from the same family, from a minority ethnic group who recently moved into the United Kingdom. Our recruitment may also have introduced or exacerbated biases. Most respondents experienced poor mental health and a high proportion had contact with police, which is not reported as typical in the literature. It is therefore possible that our respondents were not representative of the reportedly heterogeneous population who make frequent emergency calls. Nor did we achieve our recruitment target. Case management teams and other ambulance service staff did not respond to the recruitment process in the same way across organisations and regions. Our alternative recruitment approach may have helped reduce ambulance service gatekeeping to protect patients and thus given a voice to those deemed very vulnerable. It may also have enabled individuals with low trust in the ambulance service to come forward and voice their experiences.^48^, ^49^, ^50^

The number of interviews achieved was small. However, they did reflect a range of health, demographic, ethnic and economic experiences in line with our recruitment strategy. Recruiting through third‐sector organisations and social media may have reached individuals who would have been reluctant to respond to a request from an ambulance service because of poor service experience and distrust. The mixed recruitment process did provide in‐depth insight into these respondents' experiences and may in part have strengthened the study.

### 4.2 Implications for policy and practice

People who make frequent calls to emergency ambulance services have complex health needs that need both immediate and longer term action to resolve. They are also more likely to die.^12^ They usually call as a last resort, although reasons are often rooted in deeper social and economic experiences, which affect their emotional wellbeing and physical resilience. As well as the underlying health and social problems they experience, gaps in health and social care provision and barriers to accessing what is available also contribute to the picture. This paper provides rare insight into their fear and distrust of health and care services, and the mismatch between need and response that characterise the experiences of some people who make emergency calls frequently.

Service users in this study wanted to feel that their immediate needs were acknowledged and addressed with compassion and care. Many did not perceive the emergency response to be a humane one. They also needed longer term help to address deeper health and social needs affected by economic and social inequalities, as other studies have identified.^6^, ^51^

Case management has the potential to improve patient care, delivery of services and to reduce emergency demand^14^, ^52^ but how it is implemented may limit its scope to resolve people's need for help.^30^ Our study identified that some people received long‐term care and support but these interventions did not address the need as people experienced it, or stop people making frequent calls to emergency services. Many were too frightened and alienated from health and care services to identify or access treatment and support. Where services were provided, they were not in partnership with respondents or accessible to them. A case management approach must consider wider determinants of health if it is to achieve any reduction in care demand, although there is debate about how far this is realistic.^51^, ^53^ Gaps in healthcare provision also appeared to be causing callers in this study to access emergency care. The wider unmet demand in the UK for NHS general practice and OOH services is widely recognised as contributing to emergency ambulance service demand. A ‘whole system response’ including health and social care, local authorities (responsible for housing) and legal system perspectives, is needed to address issues in a coordinated and integrated way.^54^, ^55^

Supporting and treating people with complex needs involves building trust and working flexibly with individuals, yet these service users felt they had no say in the processes and decisions affecting them. Our respondents prioritised receiving judgement‐free reassurance when experiencing acute health needs. Person‐centred care is one component of multidisciplinary working aiming to improve quality of care and effective use of resources to support patients.^56^, ^57^ Those receiving case management seemed unaware that different organisations and care professionals were considering their situations, potentially making decisions about what happened to them. Nor did recipients report any changes in their experience of making frequent emergency calls. This may be due to communication issues rather than effectiveness of the intervention itself. If healthcare professionals do not engage closely enough with selected individuals to understand motivators and barriers to accessing effective healthcare, people are unlikely to feel or act differently. Services need to be provided in an accessible and relevant way to create genuine opportunities to resolve demand.^22^, ^58^ For example, mental health issues can present as an inability to follow routines or attend structured support sessions, yet be judged as failing lifestyle behaviours.^59^ Criminalising patients' actions, when their health crises jeopardise their own health and safety may not be the best approach to protecting callers or responders in the long term. Training for all staff, from call handlers to clinicians, may widen awareness of the service user's perspective to avoid any perception of exclusion and brutality and identify responses that are more effective.

### 4.3 Implications for research

Our findings reveal the feelings of desperation and powerlessness experienced by some people who call emergency services frequently. Their motivation for calling—as a last resort—indicates that current approaches to care and treatment do not resolve acute or chronic needs. Our findings show that many people feel that health and care services do not acknowledge or address their needs whilst gaps in provision exacerbate their isolation. The fact that a third of our respondents were also attended by police services indicates a need for new approaches to safe and sustained resolution. Case management has the potential to reduce demand for emergency health services.^6^ But what elements of case management work, for whom and in what circumstances is not well understood. Where the intervention is applied in the United Kingdom, it varies widely within and between ambulance services and over time.^32^ Some also interpret it as call management rather than resolving caller's needs.^29^ More research is needed about the needs and motivations of people who make frequent calls to emergency services to tailor interventions that can genuinely improve the lives of people making calls to emergency ambulance services frequently.^14^, ^15^, ^22^, ^60^, ^61^ Clarity is also required about the models and processes of case management and how it can effectively meet the needs of a diverse and vulnerable group of service users, including people of different demographic, economic and ethnic experiences.^22^ In addition, further research is needed on how to successfully recruit to research the diverse range of people who call emergency services frequently. This could support people's autonomy, which ambulance services may be gatekeeping when they control access to some patient groups^48^, ^49^, ^50^ to understand the full range of needs and experiences in this heterogeneous and unstable population.^6^, ^62^

## 5 CONCLUSION

People who make frequent calls to emergency ambulance services feel their health and care needs are urgent and ongoing.^62^ They cannot see alternative ways of obtaining help and resolving their problems. More research is needed to understand why service users make frequent calls and how to recruit them to research. Case management may address underlying and complex needs but is inconsistently applied by services, poorly understood and perceived as a trouble‐maker label by callers. Further study could explore elements of case management that can effectively address callers' perceived and actual needs.

## AUTHOR CONTRIBUTIONS

This paper was conceived and drafted by Bridie A. Evans. The manuscript received additional editorial input from Ashra Khanom, Adrian Edwards, Bethan Edwards, Angela Farr, Theresa Foster, Rachael Fothergill, Penny Gripper, Imogen Gunson, Alison Porter, Nigel Rees, Jason Scott, Helen Snooks and Alan Watkins. All authors have read and approved the final manuscript.

## CONFLICT OF INTEREST STATEMENT

The authors declare no conflict of interest.

## ETHICS STATEMENT

We received approval from the Health Research Authority (19/WA/0216) and NHS R&D permissions at all participating organisations.

## Supporting information

Supporting information.

## Data Availability

The data that support the findings of this study are available from the corresponding author upon reasonable request.
