# Supplementary material for: Experiences and views of people who frequently call emergency ambulance services: A qualitative study of UK service users
Source: Health Expect. 2023 Sep 17;27(1):e13856. doi: 10.1111/hex.13856 (PMC10726061; doi:10.1111/hex.13856)
Supplement: Supplementary file 1 — Supporting information. [file HEX-27-e13856-s001.doc]

Topic guide: Patient interviews

This research is part of a wider study trying to understand ways to manage people who often call ambulance services. People who call ambulance services sometimes don’t have an urgent medical problem. It may be better for people with less urgent needs to get the right help without having to phone 999. One of these approaches is case management. As part of this research, we want to explore the experiences of people who often call the ambulance services to enable a deeper understanding of their experiences of their illness, experience of accessing care through case management, and their thoughts on improvement.

We will ease them into the conversation and get some brief background context information on the patient, and possibly carer if present. The background information will be on age, gender, time since diagnosis, treatment, who their main care providers are, and how common unplanned hospital care is for them. After the initial introduction, open questions will be used to facilitate the person to talk about their experience using their own words. Any prompts that are used will be to encourage further discussion, understanding of the impact of interventions and ensure accurate conclusions of meaning.

The context of the study will be fully explained before the interview process starts.

1. Background questions: age (approx.), gender, time since diagnosis, treatment, who their main care providers are, and how common unplanned hospital care is for them.
2. Thinking about the calls you have made to the ambulance service, what was going on in your life?

- Why would you call the ambulance service?
- What was your experience of using the ambulance service?
- Has the response been the same or different every time?
- What is your experience of using other services (GP, pharmacists, and community support team) for this condition?
- What did you like about this/these services?

1. What do you think about case management?

- How did you feel about being on a list for case management?
- What happened when you were offered case management? Who did you have contact with?
- Have you heard people use any expressions/phrases to describe people who make a lot of calls to the ambulance service? What do you think about these? How would you like to be described?

1. What kind of support would you like to receive (therapy, welfare – housing income access to information, transition between care and services)?
